# Acute Variations in Retinal Vascular Oxygen Content in a Rabbit Model of Retinal Venous Occlusion

**DOI:** 10.1371/journal.pone.0050179

**Published:** 2012-11-20

**Authors:** Gilberto Raul Lopez Jaime, Amir H. Kashani, Saloomeh Saati, Gabriel Martin, Gerald Chader, Mark S. Humayun

**Affiliations:** 1 Doheny Eye Institute, University of Southern California, Los Angeles California, United States of America; 2 Department of Ophthalmology, William Beaumont Hospital and Associated Retinal Consultants P.C., Royal Oak, Michigan, United States of America; 3 Department of Ophthalmology, Keck School of Medicine, University of Southern California Los Angeles, Los Angeles, California, United States of America; 4 Departments of Neuroscience and Biomedical Engineering, University of Southern California Los Angeles, Los Angeles, California, United States of America; 5 Reichert Technologies, Buffalo, New York, United States of America; University of Amsterdam, Switzerland

## Abstract

**Purpose:**

To study the variation in intravascular oxygen saturation (oximetry) during an acute retinal vein occlusion (RVO) using hyperspectral computed tomographic spectroscopy based oximetry measurements.

**Methods:**

Thirty rabbits were dilated and anesthetized for experiments. Baseline oximetry measurements were made using a custom-made hyperspectral computed tomographic imaging spectrometer coupled to a fundus camera. RVO were induced using argon green laser following an intravenous injection of Rose Bengal. RVO induction was confirmed by fluorescein angiography. Retinal oximetry measurements were repeated in arterial and venous branches one hour after RVO induction and up to 4 weeks afterwards. Comparison of retinal oximetry before and after vein occlusion was made using the Student T-test.

**Results:**

One hour after RVO induction, we observed statistically significant reductions in the intravascular oxygen saturation in temporal retinal arteries (85.1±6.1% vs. 80.6±6.6%; p<0.0001) and veins (71.4±5.5% vs. 64.0±4.7%; p<0.0001). This decrease was reversible in animals that spontaneously recannulated the vein occlusion. There were no statistically significant differences in oxygen saturation in the nasal control arteries and veins before and after temporal vein RVO induction.

**Conclusions:**

We demonstrate, for the first time, acute changes in the intravascular oxygen content of retinal vessels 1 hour after RVO. These changes are reversible upon spontaneous recannulation of retinal vessels. This study demonstrates that hyperspectral computer tomographic spectroscopy based oximetry can detect physiological variations in intravascular retinal oxygen saturation. The study also provides the first qualitative and quantitative evidence of the variation in retinal vascular oxygen content directly attributable to an acute retinal vein occlusion.

## Introduction

Noninvasive measurement of hemoglobin oxygen saturation (oximetry) has been demonstrated by analysis of oxygenated and deoxygenated hemoglobin absorption spectra. [1] The earliest studies demonstrating this technique showed a difference between arterial and venous retinal oxygen saturation using photographic emulsions and multiple filter systems. [2]–[5] More sophisticated methods were later developed to allow near simultaneous measurements of a few wavelengths (usually between 2–4) to calculate oxygen saturation. [6]–[13] Other methods use rapid serial scanning with liquid tunable filters [14] or confocal scanning laser devices. [15], [16] Sophisticated calibration methods are commonly required for all non-invasive, multi-wavelength oximetry systems since the measured light depends on hemoglobin saturation as well as hematocrit, vessel size, and light scattering. [8], [17], [18] [19] An alternative method, phosphorescence quenching, measures the fluorescence of an oxygen-sensitive probe injected into the vitreous and has also been used to demonstrated retinal tissue oxygen gradients in the rat. This method is probably the most ideal measurement of *tissue* oxygen metabolism in a research setting but it is not feasible clinically [20].

The application of hyperspectral computed tomographic imaging spectrometry (HCTIS) for measurement of retinal oximetry is able to overcome some of the limitations of earlier methods. Previous animal and human studies using this method have demonstrated reliable and reproducible oximetry measurements in retinal vessels. [21], [22] HCTIS oximetry measurements show strong correlation with systemic measurements of oxygen saturation under physiologic conditions. [21] Experimental induction of retinal ischemia by reducing ocular perfusion pressure reveals reproducible variations in retinal oxygen content suggesting that vascular oxygen content fluctuates in conditions where blood flow is compromised. [21] This finding is supported by human oximetry measurements that show variations in retinal arteriovenous (AV) oxygen difference under physiological and pathological conditions. [23]–[29] For example, venous oxygen saturation in central retinal vein occlusions and branch retinal arterial occlusions is variably reduced [27], [30], [31]; however, measurements in humans can be confounded by retinal hemorrhages or nerve fiber layer edema in the early phase of disease and arteriovenous or venous-venous shunting in the later phases of disease. In many cases, confirmation of vascular occlusion by fluorescein angiography is lacking and the exact location and duration of the original occlusion is unknown. Therefore, the direct effect of the occlusive event on retinal vascular oxygen content is difficult to demonstrate and differentiate from possible secondary effects.

In order to better understand the correlation of retinal vascular oxygen content with the vascular occlusive event we have applied HCTIS oximetry measurements to a well-studied animal model of acute retinal vein occlusion. [32] In this study, we demonstrate measurements of retinal vascular oxygen content within a few hours of experimental retinal vein occlusion (confirmed by fluorescein angiography). We also demonstrate reversal of these oximetry changes after spontaneous recannulation of the vein as demonstrated by fluorescein angiography. These data demonstrate, for the first time, that vascular occlusion can directly effect oxygen content in the vascular segment that is affected by the occlusion and suggest that oximetry measurements can provide useful information about the acuity of retinal vein occlusions.

## Materials and Methods

### Animal Procedures

The rabbit retina offers numerous logistical and biological advantages for our study. The anatomy of the rabbit retina has been described extensively in previous work. [33]–[36] The vascular distribution within the rabbit retina is localized (to the medullary rays) and corresponding arteries and veins are easily imaged with conventional fundus photography. Previous studies from our lab have demonstrated that argon laser photothrombosis can induce retinal vein occlusion (RVO) and the commonly observed clinical sequelae including intraretinal hemorrhages, leakage, capillary non-perfusion, and collateral circulation. [32], [37], [38] Thirty New Zealand pigmented and albino rabbits weighing 2–3 kg were used in this study. All animal experiments adhered to the “Principles of Laboratory Animal Care” (NIH publication no. 85–23, revised 1985), the OPRR Public Health Service Policy on the Humane Care and Use of Laboratory Animals (revised 1986), and the ARVO Statement for the Use of Animals in Ophthalmic and Vision Research. Approval for these experiments was obtained from the local Institutional Animal Care and Use Committee of the University of Southern California. All procedures were performed under intramuscular anesthesia of a mixture of ketamine (50–80 mg/kg) and xylazine (5–10 mg/kg) and every attempt was made to minimize animal suffering.

### Fluorescein Angiography

Pupils were dilated with topical application of phenylephrine hydrochloride 2.5% and tropicamide 0.5% eye drops. After induction of anesthesia, an intravenous line was established in the marginal ear vein and 0.2 ml of 10% fluorescein (Akorn, Lake Forest, IL) was injected and flushed with 1 ml of normal saline. Sequential fundus photographs with fluorescein filters were taken using a Spectralis HRA+OCT (Heidelberg Inc, Heidelberg, Germany) immediately after fluorescein injection. Late photographs were taken at 3 and 5 minutes. Fluorescein angiography was performed at baseline and again after induction of vein occlusion at 1 hour, 1 week, and otherwise as indicated in the figures.

### Hyperspectral Computed Tomographic Imaging Spectroscopy

Hyperspectral images were obtained with a custom-made hyperspectral camera attached to the accessory port of a standard, commercially available Zeiss FF450 IR fundus camera as previously described. [21] Briefly, the HCTIS can acquire approximately 76 spectral bands (450–700 nm) within the duration of a standard fundus photograph. Images are acquired by a digital camera and stored on a computer using standard image acquisition software. The calculation of intravascular oxygen content (oximetry) was performed using a modified Lambert-Beer approximation of the vessel optical density as described elsewhere. [21] Retinal oximetry was modeled as a least-squares approximation of 28 wavelengths from the oxy- and deoxyhemoglobin spectra. [21] *In vivo* calibration and detailed description of the oximetry methods have been reported elsewhere. [21], [22] For each RVO induced in the temporal retinal circulation, control images were obtained from the non-occluded nasal retinal circulation in the same eye. HCTIS images were performed at baseline and again after induction of vein occlusion at 1 hour, 1 week, and otherwise as indicated in the figures. Results are displayed as pseudocolored oximetry maps where red represents 100% oxygen saturation and blue represents 0% saturation.

**Figure 1:**
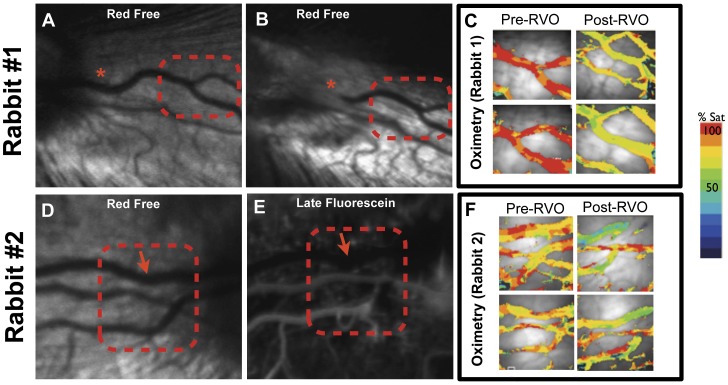
Intravascular oxygen content decreases after retinal vein occlusion. Two examples of retinal vein occlusion and oximetry images of retinal vessels from two different rabbits before and after retinal vein occlusion (RVO). (A) Red free image of the normal rabbit retinal vessels before and (B) one hour after retinal vein occlusion. The red asterisks indicates the location of the occlusion. Note the absence of blood column after onset of occlusion. (C) Two examples of pre-RVO and post-RVO pseudocolored oximetry images from red dotted area showing decreased intravascular oxygen content one hour after RVO. (D) Red free image of the normal rabbit retinal vessels before RVO and (E) late phase fluorescein angiogram of same area one hour after retinal vein occlusion. The arrow marks location of non-filling vessel which is difficult to see in late phase fluorescein image. Red dotted area indicates area of pseudocolored oximetry images in panel F. In both panel C and F, note the decrease in the vascular oxygen content after venous occlusion.

### Retinal Vein Occlusion (RVO) Induction

The detailed methods of RVO induction have been described in previous publications and were replicated in this study. [32], [39] In twenty rabbits RVO induction was achieved by multiple applications of 532 nm argon green laser (Iris Medical Oculight Glx IRIDEX Corporation, Mountain View, CA) to the retinal vein a few seconds after intravenous injection of Rose Bengal (40 mg/kg Sigma-Aldrich Inc). Typically, the primary retinal vein was occluded within 12 disc diameters of the disc margin. The laser spot size was approximately 125 m (approximately the size of the retinal vein caliber), laser power was between 150300 mW and the duration of each pulse was 0.5 seconds. Ten to 25 shots were applied to a single location on the retinal vein until occlusion was clinically visible. Confirmation of RVO was made with fluorescein angiography 1 hour after clinical visualization of RVO. Care was taken to obtain accurate focus of the aiming beam on the vessel by adjusting the distance between the lens and the eye. The occlusion was considered complete if there was no evidence of venous filling in the mid-phase of the angiogram along the target vessel in comparison with the nasal vein within the same retina (that served as a control). In many cases, there was evidence of slow reverse flow in major veins and dye leakage into the retina in later phases. Supplementary Movie 1 and 2 (Movie S1 and S2) illustrates an example of the rabbit circulation at baseline and after RVO induction, respectively.

In five additional rabbits, the laser application was intentionally applied to the nerve fiber layer as a control for the effects of laser on the surrounding tissue rather than on the vessel directly. In another 5 additional animals, the laser application was performed in exactly the same manner except 5–10 shots were applied and no RVO was observed on fluorescein angiography. These experiments were designed to control for the possible non-specific effects of laser on the vessels and surrounding tissues. In all cases, animals were sacrificed at the indicated times using standard intracardiac or intravenous injection of 120 mg/kg sodium pentobarbital to induce cardiopulmonary arrest.

### Statistics

Data are presented as standard deviations of the mean unless indicated otherwise. Comparisons were made between the mean oximetry measurements of the retinal artery and vein before and after RVO induction. A paired, two-tailed Student t-test was used for analysis of the oxygen saturation difference between all pairs of vessels. All statistical testing was performed using GraphPad Prism, version 5 (GraphPad Software, La Jolla CA, USA).

## Results

**Figure 2:**
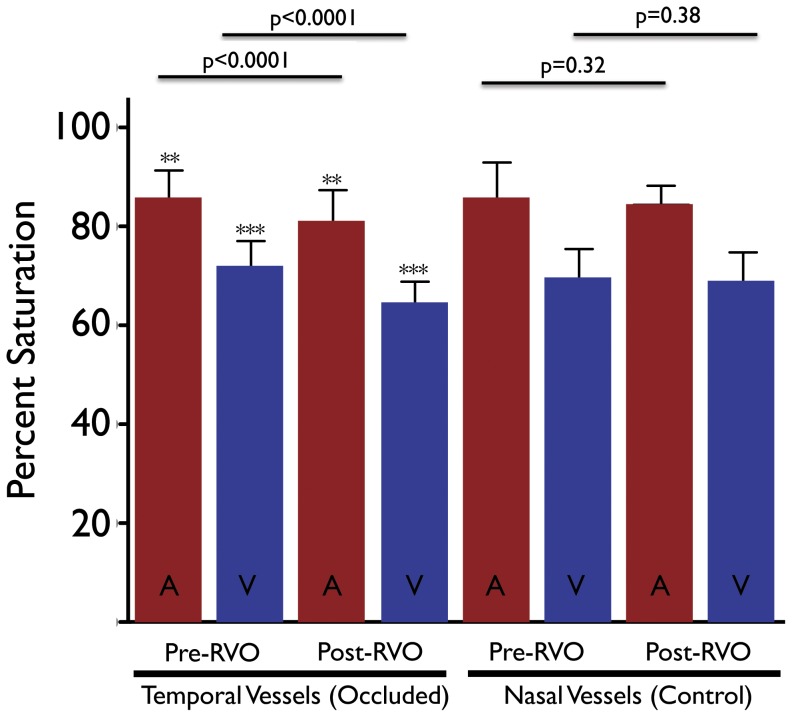
Summary of arterial and venous oximetry measurements from rabbits before and one hour after RVO. Red bars indicate arterial measurements. Blue bars indicate venous measurements. RVO induction was performed in the temporal veins only. Nasal vessels were used as an internal control for the variability in oximetry measurements. There was a significant decrease in the retinal vascular oxygen saturation of the occluded vessel before and after RVO. There was no significant difference between the retinal vascular oxygen saturation of the control vessels before and after RVO.

**Figure 3:**
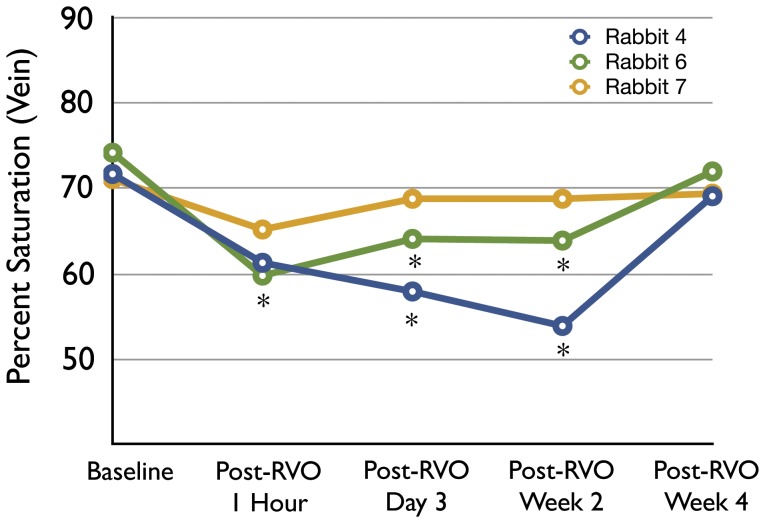
Increase in the retinal venous oxygen saturation after spontaneous recannulation of the retinal vein in 3 rabbits. RVO was successfully induced on day 0 (baseline) and resulted in a significant decrease in venous oxygen content compared to baseline through 2 weeks of follow-up in rabbits 4 and 6 (asterisk indicates p<0.001; Each point is the average of multiple vessel segment measurements in one rabbit at one imaging session). The standard deviation of the data points ranges from 4–8% however, error bars have been omitted for clarity. Only partial RVO induction was achieved in rabbit 7 on day 0 as determined by fluorescein angiography. Over the succeeding few days this completely recannulated. The retinal venous oxygen content of the partially occluded vessel in rabbit 7 was not significantly lower than baseline at any point.

Twenty rabbits with RVO were imaged using the HCTIS previously described. [21] Figure 1 illustrates representative red free, fluorescein angiogram and pseudocolored oximetry images from two rabbits before and one hour after RVO induction in the temporal retinal vein. Movies S1 and S2 illustrate an example of the rabbit circulation at baseline and after RVO induction, respectively. In all animals, the nasal retinal vein of the same eye was used as a control for each animal. The mean baseline temporal arterial (A_ox_) and venous (V_ox_) oxygen saturation was 85.1±6.1% and 71.4±5.5% respectively before RVO induction (n = 20). One hour after RVO induction, mean A_ox_ and V_ox_ was 80.6±6.6% and 64.0±4.7%, respectively (n = 20). This represented a statistically significant decrease in both values from baseline (p<0.0001). For nasal control vessels, baseline mean A_ox_ and V_ox_ measurements were 85.2±7.6% and 69.2±6.2% respectively (n = 20). There were no significant changes in the retinal oximetry measurements of the nasal vessels after temporal RVO induction (Figure 2). Similarly, there was no significant difference between the temporal and nasal A_ox_ or V_ox_ measurements before RVO induction (Figure 2). In a limited number of animals (n = 3) in which the RVO spontaneously recannulated (as demonstrated by serial fluorescein angiography) we observed that the venous oxygen saturation returned to baseline levels at the time of recannulation (Figure 3).

**Figure 4:**
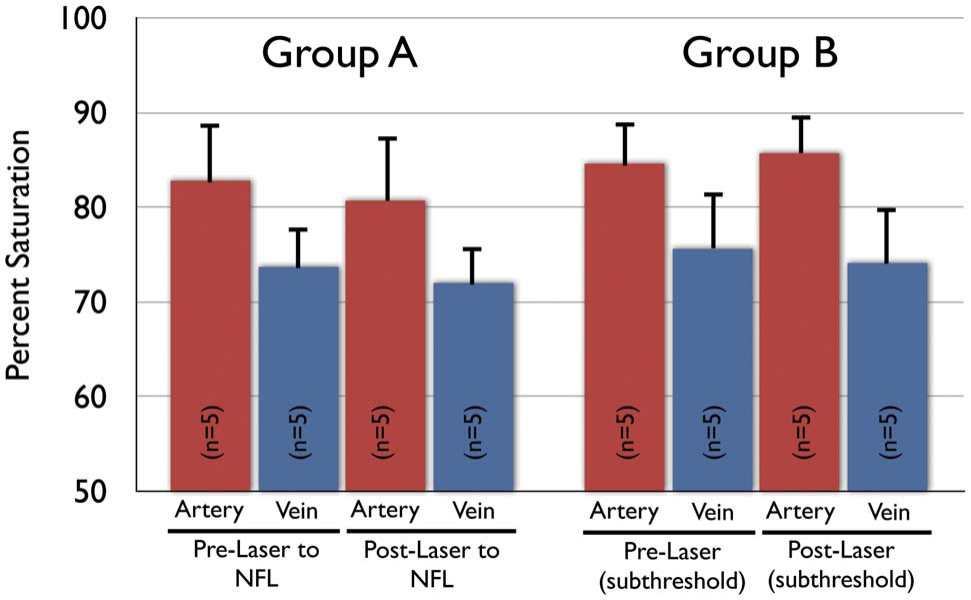
Control experiments were performed to determine the effect of laser application independent of RVO induction. Group A animals received laser to the tissue surrounding the retinal vein and did not show RVO on fluorescein angiography. There was no significant difference between oximetry measurements of the retinal vessels before and one hour after laser application in Group A. Group B animals received sub-threshold laser to the retinal vein and did not achieve RVO on fluorescein angiography. There was similarly no significant difference between oximetry measurements before and one hour after this subthreshold laser application in Group B.

In order to control for the effects of laser application on surrounding tissue and the non-specific effects of laser application in general we performed two additional experiments. In one series of experiments, laser application was applied with the same parameters as in standard RVO induction but the laser was aimed immediately adjacent to the vein (i.e. laser applied to nerve fiber layer) for 10–20 applications (Figure 4; Group A). Fluorescein angiography and clinical observation demonstrated no RVO in these cases. Baseline mean A_ox_ and V_ox_ in these animals were 82.8±6.7% and 73.7±4.4% before laser, respectively. One hour after laser, mean A_ox_ and V_ox_ were 80.7±7.2% and 72.0±4.0%, respectively (n = 5). These were not statistically significant changes in A_ox_ (p = 0.31) or V_ox_ (p = 0.13).

In another series of control experiments, laser was applied to the vein but at lower power than in those with successful RVO induction (Figure 4; Group B). In these cases, the effects of laser on the vasculature and surrounding tissue may be assessed without actual formation of RVO. Fluorescein angiography and clinical observation demonstrated no RVO in these cases. Baseline A_ox_ and V_ox_ were 84.6±5.9% and 75.6±6.3% respectively (n = 5) in these controls. One hour after laser, mean A_ox_ and V_ox_ were 85.7±5.5% and 74.1±6.4%, respectively (n = 5). These were not statistically significant changes in A_ox_ (p = 0.32) or V_ox_ (p = 0.13).

## Discussion

In the present study, we demonstrate the variation of retinal vascular oxygen content in an acute experimental model of retinal vein occlusion using hyperspectral computed tomographic imaging spectroscopy. [32] Both the animal model and oximetry method have been previously validated. [21], [22], [32], [39] We are able to demonstrate for the first time that retinal vein occlusion does in fact induce an immediate decrease in retinal vascular oxygen content. We also demonstrate reversal of these oximetry changes with spontaneous recannulation of the vein as demonstrated by fluorescein angiography. These data demonstrate that vascular occlusion can directly effect oxygen content in the affected vascular segment and suggest that oximetry measurements can provide useful information about the acuity of RVO.

In the retinal literature, it has generally been presumed that the primary mechanism of vascular occlusive damage is retinal hypoxia. This has not been demonstrated directly for a number of reasons. First, the development of non-invasive oxygen measurement methods (oximetry) has only made intravascular oxygen measurements possible in the last several years. [1], [21] Measurements of intraretinal oxygen tension are still not feasible because of the invasive nature of this method. [20] Second, measurements of spontaneous vascular occlusions, such as those in human CRVO and BRVO, are almost always confounded by lack of knowledge regarding the temporal onset and spatial distribution of the occlusive event. [27], [30] Patients with spontaneous vascular occlusive events may present anywhere from a few days to several months after the onset of the symptoms. Therefore measurements of intravascular oxygen content in human subjects are confounded by compensatory responses to the occlusive event such as venous-venous or arterial-venous shunting, formation of collateral vessels and neovascularization. [40] Another potential confounder is spontaneous recannulation of the occlusion. Third, in cases where oximetry measurements can be made in the acute setting of a vein occlusion in humans (usually within the first few days of the event), the measurements are still likely to be confounded by the intraretinal hemorrhages and cotton-wool spots that obscure the underlying vessels and retina. Therefore, there is significant advantage in studying acute changes in retinal oxygen content after an occlusive event in an animal model where the event can be induced and studied immediately.

This study suggests that in the absence of retinal venous flow secondary to thrombus formation, the oxygen saturation within the upstream retinal veins and arteries decreases significantly (Figure 2). Specifically, after induction of RVO we observed a 7.4% decrease in the oxygen saturation of the veins (p<0.001) and 4.5% decrease in the arteries (p<0.001). This change was reversible in those animals that showed spontaneous recannulation (Figure 3). This suggests that the decrease in saturation is causally related to the obstruction of flow demonstrated by fluorescein angiography. In our experiments, the absolute value of the decrease in oxygen saturation varies from animal to animal. Careful analysis of the fluorescein angiograms suggests that retrograde filling through collateral channels may account for this variability. Similar variability has been demonstrated in humans although the magnitude of variability is much greater in humans [24], [27].

It is notable that the results presented here demonstrate a decrease in oxygen saturation in the retinal artery despite the absence of a thrombus occluding the artery. Although there can be retrograde propagation of the thrombus affecting both the artery and the vein, this is unlikely to occur within the 1 hour time point of our measurements. [32] Alternatively, complete occlusion of the venous outflow would be expected to decrease arterial flow even without retrograde propagation of thrombus. Clinically, the appearance of numerous hemorrhages in patients and animal models of RVO are explained by this retrograde congestion of the vasculature. [32], [40] The decrease in oxygen saturation observed in these experiments would support either possibility.

There are a number of confounders that may have affected our experimental measurements. Specifically, the application of argon laser to the retinal vessels may have additional non-specific effects on vascular permeability and tissue metabolism in addition to causing thrombus formation. To control for these possibilities, we performed two series of experiments in which laser was applied either to the tissue surrounding the retinal vein (rather than the vein directly) or applied to the vein without causing an occlusion. In the former experiments, the application of laser to the tissue surrounding the vein controls for any non-specific effects of laser damage on retinal tissue. This damage may alter retinal metabolism and overall tissue viability which would alter oxygen demand in the vicinity of the vessel. In the latter experiments, the application of laser to the vein at lower power does not cause thrombus formation but still replicates damage to the retinal vessels and vascular permeability. In neither of these control scenarios did we observe significant changes in the intravascular oxygen measurements (Figure 4). This suggests that the decrease in intravascular oxygen content that is demonstrated in occluded vessels is not a non-specific effect of laser on the tissue but rather a direct effect of thrombus formation and obstruction of flow.

Oximetry measurements are inherently confounded by many factors including media clarity, vessel size, retinal pigmentation and scatter. [1], [21] Previous work has demonstrated the advantages of the hyperspectral computed tomographic imaging method used in this study. In the context of this paper, “hyperspectral” refers to the simultaneous acquisition of spectra from 450–700 nm with ∼4 nm spectral resolution using a two dimensional diffraction grating and computed tomographic imaging algorithms. [41], [42] This method provides excellent spectral, spatial and temporal coregistration *in vivo* compared to devices that rely on spectral or spatial scanning. [21] This hyperspectral system is mounted on a commercially available fundus camera and demonstrates reliable and reproducible high resolution measurements of hemoglobin oxygen saturation within retinal microvasculature *in vivo*. [21] These measurements are resolved over the duration of standard fundus flash photography and within vessels as small as ∼50 microns wide. The correlation of oximetry measurements with vascular occlusion in this paper would seem to support the accuracy of and reliability of this method.

For the first time, the present findings illustrate that retinal venous occlusion directly correlates with decreased retinal vascular oxygen content in both arteries and veins. In a mouse model of subdermal microvascular thrombosis, Wankhede et al have also found decreased oxygenation in the venules of the dorsal skin fold after chemical induction of thrombosis in vivo. [43] These findings support the general notion that vascular occlusion cause tissue damage via hypoxia. Quantitation of retinal vascular oxygen content in humans may have important implications by allowing the clinician to assess the severity of hypoxia rather than rely solely on the classical clinical signs of vein occlusion.

## Supporting Information

Movie S1 **Fluorescein angiogram of normal rabbit retina.** Movie demonstrates complete transit for one eye of healthy adult rabbit.
(AVI)

Movie S2 **Fluorescein angiogram of same rabbit as in S1 immediately after induction of retinal vein occlusion in temporal retinal vein.** Movie demonstrates a complete transit with nonperfusion of the majority of the temporal medullary streak.
(AVI)
